# Building the Prestige of *Archivum Immunologiae et Therapiae Experimentalis*: From a Little Known to an Internationally Recognized Journal

**DOI:** 10.1007/s00005-018-0529-3

**Published:** 2018-10-16

**Authors:** Hubert Krotkiewski, Andrzej Górski, Michał Zimecki

**Affiliations:** Hirszfeld Institute of Immunology and Experimental Therapy, Polish Academy of Sciences, Weigla 12, 53-114 Wrocław, Poland

**Keywords:** AITE, Ludwik Hirszfeld, Impact factor, Editorial policy, Immunology, Experimental therapy

## Abstract

*Archivum Immunologiae et Therapiae Experimentalis* (AITE) was founded in 1953 by Ludwik Hirszfeld, a world famous Polish physician and scientist in the field of microbiology and immunology. Initially, AITE was published in Polish, but within a few years, it changed to English to increase the range and number of international readers. In its over 65 year history, AITE has had several Editors and a number of Publishers. In the period 1977–1991, AITE was listed in the system of scientific information Current Contents/Life Sciences, but for several years, its impact on the international readership of the Journal was negligible. The political and economic crisis in Poland in late 1980s led to serious delays in printing of successive AITE issues, so the Journal was removed from the Current Contents. Year 1991 was a turning point for the Journal, guided since then by prof. Dubowska-Inglot, who changed its image and format, and allowed acceptance of review articles. In 1999, prof. Górski became the Editor-in-Chief, giving a new impulse for further development of the Journal. In a consequence, AITE was accepted to Science Citation Index Expanded (in 2001) and to Institute for Scientific Information Master Journal List (in 2002). Eventually, AITE has evolved to become a truly international, multidisciplinary journal, publishing original articles, and reviews relating to basic and clinical immunology, experimental therapy, immunogenetics, transplantology, microbiology, immunochemistry, as well as bioethics. Currently, AITE is cited in a number of major scientific information databases. Since 2011, the Journal is published by Springer Publishing House, it has achieved international recognition with its latest impact factor (for 2017) of 3.018. AITE, whose Editors are professors of Hirszfeld Institute of Immunology and Experimental Therapy, strengthens the status and position of the Institute as one of the leading scientific institutions in Poland.

## Founding, Goals, Editors and Publishers

**Fig. 1:**
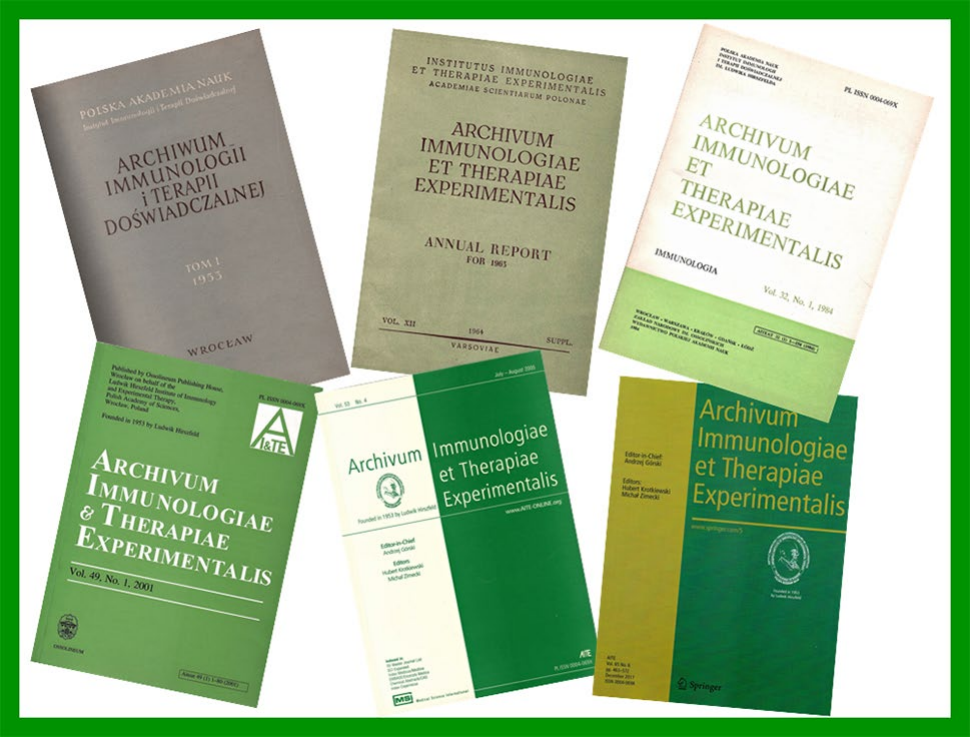
The covers of the printed versions of AITE, chronological order

*Archivum Immunologiae et Therapiae Experimentalis* (AITE) was founded in 1953 in Wrocław (Poland) by Ludwik Hirszfeld, a world famous physician, serologist and immunologist. At the time in the postwar Poland, a great demand existed for establishment of a new journal due to a remarkable progress in the field of immunology and related medical sciences. The Journal was initially edited in Polish, but after some years, English was introduced for all articles to increase the range and number of potential international readers. AITE in its 65-year history had several Editors-in-Chief (in a chronological order): Henryk Makower (1953–1955), Stefan Ślopek (1956–1987), Jerzy Giełdanowski (1988–1991), Anna Dubowska-Inglot (1991–1998) and since 1999 until now—Andrzej Górski. The Journal was initially published by Polish Scientific Publishers (PWN, Warsaw, in 1953–1955), then by Polish Medical Publishers (PZWL, Warsaw, in 1956–1977), Ossolineum (Wrocław, in 1978–2003), Medical Science International (Warsaw, in 2004–2005), Birkhäuser (Basel, in 2006–2010) and finally Springer (Basel, from 2011 till now). Figure 1 presents the changing covers of the printed versions of AITE.

## Early Years of AITE

At the beginning AITE was published solely in Polish, and from 1961, the articles are in English. Although AITE was added to Current Contents/Life Sciences (CC/LS) system in 1977, for several years its impact on the international readership of the Journal was low. The political and economic circumstances in Poland in the late 1980s led to nearly a year delay in printing of AITE issues, which finally resulted in deselecting from CC/LS in November 1991. The Journal’s condition was poor—the scientific value of articles, as well as the reviewing procedures, was not adequate; in addition, the bureaucracy and poor management of the then publishing house negatively affected editorial work. In consequence, the impact factor (IF) for 1988 was the lowest in AITE history (0.104). Table 1 depicts some IF values of AITE in the years 1980–1988.

**Table 1:** The impact factor of *Archivum Immunologiae et Therapiae Experimentalis* in the years 1980–1988

Year	IF
1980	0.258
1981	0.250
1982	0.119
1985	0.220
1987	0.211
1988	0.104

## New Times, New Challenges, First Achievements

In 1991, prof. Anna Dubowska-Inglot was appointed Editor-in-Chief; she offered the positions of deputy editors to prof. Hubert Krotkiewski and prof. Michał Zimecki; subsequently, Ms Anna Steć, a very experienced secretary, became editorial assistant. At that time, an important reconstruction of the Journal was undertaken by (1) changing its size to A4, (2) introducing color figures and review articles (apart from original ones) and (3) introducing a two-column format. Fortunately, the respective issues of the Journal started again to be printed duely on time. In 1999, after passing away of prof. Dubowska-Inglot, prof. Andrzej Górski was appointed a new Editor-in-Chief, giving a further impulse to improve the quality of the Journal. A new approach to achieve a higher scientific status and international position of AITE involved the procedure of inviting review articles from renowned potential authors. In 2000, the IF of AITE was still modest (0.413) although four times higher than in 1988. AITE started to be much better recognized on the international publishing market. Taking the chance of a higher influx of review articles, AITE was able to publish, in collaboration with Kluwer Academic Publishers, two monothematic books: *Autoimmunity* and *Inflammation* (Górski et al. 2001a, b). Thanks to continuous efforts of the Editorial Team, the Journal was accepted in 2001 by Institute for Scientific Information (ISI, Philadelphia) to Science Citation Index Expanded database (IF 0.587) and in 2002 to the ISI Master Journal list with IF 0.793.

## Attaining International Position

After collaboration with Ossolineum AITE was taken up in 2004–2005 by Medical Science International (Warsaw), which used more up-to-date editorial technologies, this change resulted in higher IF 1.00 in the year 2005. Such progress attracted the interest of Birkhäuser Publishing House (at that time filia of Springer), which we signed a publishing agreement with starting 2006. In consequence, in the years 2006–2010 AITE was published by Birkhäuser. Thanks to association with this professional, international publishing house, the Journal accomplished a tremendous progress by achieving IF 2.385 in 2010. In 2011 we started the collaboration with Springer which brought further increase of AITE citation (IF 3.176 in 2014). This constant increase of AITE scientific level let the Journal rank three times first: in 2011 (IF 2.541), 2013 (IF 2.818) and 2014 (IF 3.176) among Polish scientific journals, present in ISI Master Journal list. Following a brief decline in IF value in the years 2015–2016, AITE received IF 3.018 (2017), which is close to the highest value in the history of the Journal (IF 3.176 in 2014). We are proud to be successful in continuously attracting authors from prestigious universities, like: Harvard, Oxford, Yale, Tokyo, Athens, Houston, Hong Kong, Edinburgh, Osaka, McGill, Karolinska Institutet and many others. AITE articles are being cited in the leading scientific journals, such as: Science, Nature, Journal of Immunology, Journal of Experimental Medicine, PNAS, Oncotarget, Frontiers in Immunology, Frontiers in Microbiology, Scientific Reports, PLoS One, etc. In 2011 the Editorial Manager (EM) system was introduced to our editorial practice which is a rule in the most modern scientific journals. The system substantially facilitates editorial procedures and manuscript processing. AITE is currently cited in the following basic science information systems: Science Citation Index Expanded (SciSearch), Journal Citation Reports/Science Edition, PubMed/Medline, SCOPUS, EMBASE, Chemical Abstracts Service (CAS), Google Scholar, CSA, CAB International, Academic OneFile, AGRICOLA, Biological Abstracts, BIOSIS, CAB Abstracts, Gale, Global Health, INIS Atomindex, International Bibliography of Book Reviews (IBR), International Bibliography of Periodical Literature (IBZ), OCLC, Journal Citation Reports/Science Edition, SCImago and Summon by Serial Solutions.

## Policy and Editorial Work: Challenges, Traps, Surprises

The policy of AITE assumes recruitment of the best quality manuscripts irrespectively of affiliation, country of origin and scientific output of a potential author, however, manuscripts from renowned authors and institutions have our preference. First, the manuscripts are subjected to the editorial in-house evaluation and these outside the scope or of lower priority are not processed further. The Editors, as a rule, do not accept case reports. As a next step, the manuscripts are subjected to peer review by at least two foreign experts in the field, although some articles require evaluation by more referees. Invited articles are published free of charge. The Journal accepts both original and review articles, although from Editorial Office point of view, the latter are preferred due to a higher readership and citation. In practice, the number of reviews and original articles per issue are roughly comparable (1:1). The policy of AITE also stipulates that new members of the Advisory Board are recruited from new authors and their exceptional contribution to the scientific level of the Journal is a requirement. The list of Advisory Board is a subject to modification every 3 years.

The gaining of the good quality articles is a serious challenge. Apart from spontaneously submitted manuscripts, of which many may not meet the Journal’s requirements, we try to focus on a category of “invited reviews”. Such invitations are usually aimed at authors published in high-quality journals, for example, *The Journal of Immunology*. Recently a remarkable increase of the number of new journals is evident, mostly published online, which leads to a dramatic competition on the market. Luckily, these newly generated journals are not attractive enough due to lack of established IF and absence, at the beginning, in the scientific information systems. In this respect, the tradition of AITE (founded in 1953) and its excellent history of attaining the international status, represent an advantage for the Journal. About 50% of the articles, published in AITE, are currently (2018) accessible to readers free of charge.

Due to a high number of the good quality journals in the category Immunology the position of AITE in this classification (the journals, for ranking purposes, are divided into four groups/quartiles within one category) varied depending on IF gained in a given year. AITE ranked in the years 2010–2012 in quartile Q3, in quartile Q2 in the years 2013–2014, in quartile Q3 in the year 2015 and in quartile Q4 in the year 2016. The IF 3.018 for the year 2017 moved AITE to quartile Q3 again. It is also obvious for us that because of high competition on the market recently we may observe serious shifts in a position of previously leading journals, e.g., *The Journal of Immunology* suffered a drop in its IF from 7.065 (in 2001) to 4.539 (in 2017). Although new attractive titles in the field of immunology emerged, due to a constant progress AITE found a firm place among important immunological journals (IF in parenthesis), such as: Immunobiology (2.720), Cellular Immunology (3.172), International Immunopharmacology (2.956), PLoS One (2.806), Acta Pharm Sinica (3.223), Basic and Clinical Pharmacology and Toxicology (3.176) or Scandinavian Journal of Immunology (2.256). These comparisons clearly show a leading position of AITE among immunological journals in Central and Eastern Europe (Groneberg 2018).

The most difficult part of our editorial work is a proper selection of articles which would be interesting to the readers. This is reasonably easy for some types of review articles but sometimes we can not foresee a potential “success” of a given article. A case in point: it happened that we published several articles from one research group and they were very poorly cited causing our objection to accept another manuscript from the same laboratory. In addition, the opinions of the reviewers regarding the newest paper were unpromising. However, to our surprise, the article had quite high citations. Another important issue regarding acceptance of articles for publication is that it turned out to be risky to publish articles from conferences presenting subjects not directly associated with the publishing scope of AITE. In these cases the articles were not at all or very poorly cited which, in consequence, negatively affected the Journal’s IF.

**Fig. 2:**
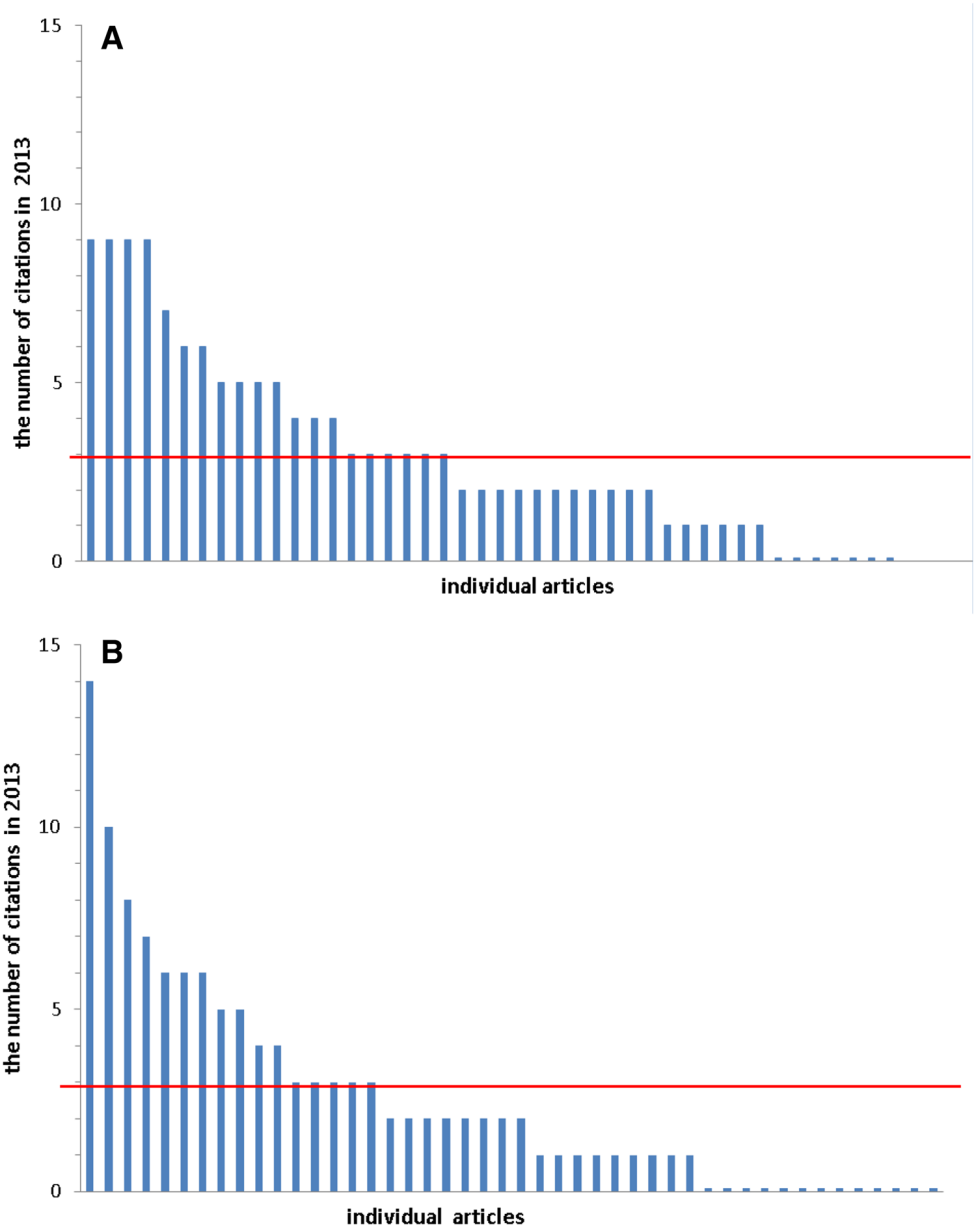
Two sets of citations, used to calculate IF for the year 2013; **A** citations received for 43 articles, published in AITE in the year 2011 (vol. 59), 7 articles (16.3%) had no citations; **B** citations received for 45 articles, published in AITE in the year 2012 (vol. 60), 13 articles (28.9%) had no citations. Red line indicates IF value calculated for the year 2013

It is of course clear that the significance of IF value for a scientific journal should not be overestimated. A widely spread opinion prevails that IF does not actually reflect the real scientific value of a journal (Callaway 2016; Ketcham 2008) and what really counts is a citation of a particular article (Falagas and Alexiou 2008; Ketcham 2008). As Eugene Garfield, a “father” of IF system, says “impact factor is not a perfect tool to measure the quality of articles but there is nothing better and it has the advantage of already being in existence and is, therefore, a good technique for scientific evaluation” (Garfield 1999). In everyday editorial work we are aware of all doubts mentioned above. It is characteristic for all journals, as well as for top-cited journals, like Nature and Science (Callaway 2016), that the citation distribution of articles is highly out of proportion, i.e., majority of articles are cited below the journal’s IF; for comparison see the relevant data for AITE (Fig. 2).

Nevertheless, in the Polish Ministry of Science and Higher Education (Pilc 2008) system of evaluation of scientific institutions IF values of articles published by the scientists, employed in an institution, correspond to so-called “ministry points”. As a consequence, a sum of the points, representing scientific output of an institution and its other relevant achievements, determines its position in the ranking and, what is most important, is reflected by adequate funding. High value of AITE’s IF is an important factor, contributing to the Ministry’s funding of the Institute, which is one of the well known scientific institutions in Poland (Zimecki 2004).

## Ethical Standards, Self-Citations

AITE Editors pay particular attention to maintaining high ethical standards of the Journal and thus the Editors decided not to publish their own articles in AITE. Solicitation, expressed by Editorial Office, to cite AITE articles by the authors of accepted papers, (which is an unfortunate practice in some journals), is totally unthinkable. In consequence, the percentage of self-citations in AITE articles is amazingly low (see Table 2) in contrast to the mean self-citation rate equal to 12.41% (McVeigh 2002).

**Table 2:** Examples of self-citations (mean values), calculated for the period 1990–2018 (data based on Web of Science core collection)

AITE	1.4%
Immunology and cell biology	2.1%
International immunology	2.4%
Cellular immunology	2.6%
Journal of experimental medicine	2.6%
Clinical and Experimental Immunology	3.6%
Autoimmunity	4.1%
Pharmacological reports	8.5%
Frontiers in immunology	10.7%
Journal of physiology and pharmacology	12.8%

The Editorial Office is also aware of potential plagiarism, and therefore, the submitted manuscripts are monitored using an anti-plagiarism computer program. Ethical responsibilities of the authors are included in the Instructions to authors and require a statement of: (1) novelty of the presented results not published before; (2) potential conflicts of interest; (3) agreement of local Ethics Committee regarding the research involving human participants and/or experimental animals and (4) informed consent of patients and volunteers donating biological material. The *Journal Citation Reports* (JCR), published by Clarivate Analytics since 1975, monitor the journals towards unexplainable high citations and, in the cases of dishonest procedures of acquiring citations, remove those journals from JCR databases for 1 year. AITE, in its 65 years history, has always been blameless regarding this issue.

## Present Status of the Journal, Perspectives

In the last 26 years the scientific value of AITE has acquired good international level. Our Advisory Board currently consists of 39 members, 26 of whom (67%) come from the foreign scientific institutions. To complete the current, overall image of AITE below we present some characteristic data:

### Reviewing Process

All manuscripts, submitted to AITE and accepted by Editors, are subject to peer review procedure. As a rule we look for reviewers abroad and pay due attention to their thorough opinions and subsequently to Authors’ responses, who are mostly satisfied with these comments. This is reflected in their positive attitude, e.g., “The reviewers’ comments have been most helpful to improve the quality of our manuscript”, “Thank you very much for this opportunity to publish in AITE”, “We express our gratitude to Reviewers for their comments that helped us to improve the quality of the article”. We fully understand that thanks to the Reviewers’ work the scientific quality of the Journal constantly improves.

### Rejection Rate

Table 3 presents rejection rate (number of rejected articles compared to all submitted manuscripts, expressed in percentages).

**Table 3:** Rejection rate regarding manuscripts submitted to AITE

Year	Rejection rate (%)
2006	27
2007	24
2008	19
2009	27
2010	48
2011	26
2012	33
2013	41
2014	30
2015	42
2016	36
2017	53

AITE publishes yearly ca 45–50 articles; from the Table 3 it is evident we reject approximately half of the submitted manuscripts, or less. The better the journal is, the more manuscripts it is able to reject.

It should be noted that some 25 years ago the influx of the manuscripts being very poor, the Journal did not reject a single article. Nowadays the number of manuscripts submitted is considerably higher and we have a real choice as to which manuscripts should be published.

### Citations of AITE Articles

As mentioned above, citation is the best measure of the scientific value of an article and the journal, where it was published. The best-cited AITE articles are as follows (based on Web of Science Core Collection data; October 19th, 2018):

- 179 citations—Zhang et al. (2009),
- 147 citations—Shi and Wei (2007),
- 147 citations—Varnum and Ikezu (2012),
- 142 citations—Bujak and Frangogiannis (2009).
- 136 citations—Kobayashi et al. (2005).

AITE, in its 65 years of publishing history, had so far 13 articles cited more than a hundred times and 43 articles were cited at least 50 times. When an article is cited in a scientific publication, it is a clear indication that it was interesting and significant for the scientific world. In consequence, individual citations influence IF of a journal.

### Full-Text Article Requests

Another measure of interest in a journal’s articles, although more relative, are full-text down loadings from the website of a journal. Based on AITE Publisher’s Report (2017) these full-text article requests in the recent years were as follows:

- in 2010 40,028 requests
- in 2011 44,560 requests
- in 2012 45,443 requests
- in 2013 55,413 requests
- in 2014 49,484 requests
- in 2015 52,674 requests
- in 2016 86,744 requests
- in 2017 109,159 requests

In our opinion the number of the full-text article requests is high and means that AITE articles are interesting to the readers. Moreover, the number of these down loadings is constantly increasing, which is very promising for the Journal.

### Impact Factor

**Fig. 3:**
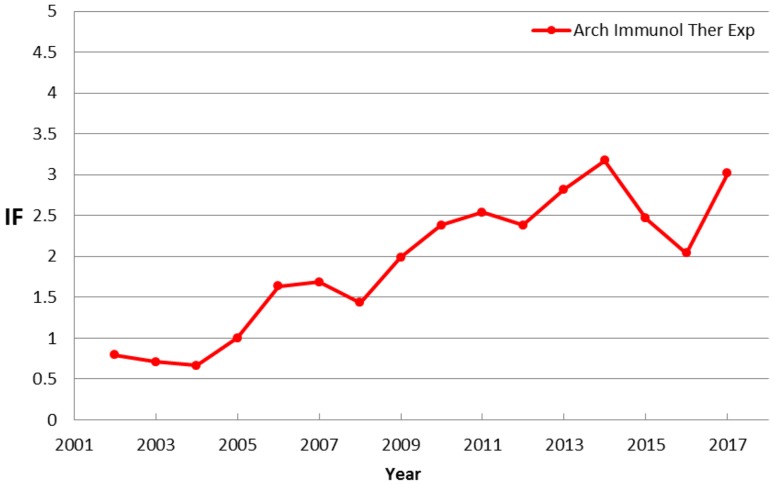
Diagram presenting IF values of AITE in the period 2002–2017

Figure 3 presents IF values of AITE in the period 2002–2017. As can be seen, within 15 years the IF calculated for AITE has grown from 0.793 (2002) to 3.018 (2017). We can see a very dynamic and continuous development of the Journal, which directly means acquiring the good quality manuscripts. Such a diagram can be treated as a relative ID of a journal, because it shows an important final publication parameter, reflecting a response of a market to a real scientific value of the journal.

### Distribution

Currently AITE is published in two forms: printed version (ISSN 0004-069X) and electronic version (ISSN 1661–4917); the latter can be reached under the address http://www.springer.com/5. The navigation bar includes Online first articles, where there is a set of papers accepted for publication but not yet printed, with a given DOI number which already enables citation.

Editorial Office

- Editor-in-Chief Andrzej Górski since 1999
- deputy editor Hubert Krotkiewski since 1991
- deputy editor Michał Zimecki since 1991
- editorial assistant Anna Steć since 1990

## Last but Not Least

Our plans for the nearest future assume continued and fruitful collaboration with Springer Publishing House. Our efforts will also be aimed at achieving a complete coverage of the Journal’s budget (both the costs of publication and personal costs) by adjusting the page-charges to the advancing scientific status of the Journal, which we are determined to maintain on the highest possible level.

In December 2016 the Editorial Team of the Journal received a scientific award of the President of Polish Academy of Sciences, professor Jerzy Duszyński, for “earning a notable international prestige for the Journal due to the high publishing standards over many years of its publication”. We do hope this award will strongly raise the international position of the Journal in the coming years.
